# Role of vitamin K2 in preventing the recurrence of hepatocellular carcinoma after curative treatment: A meta-analysis of randomized controlled trials

**DOI:** 10.1186/1471-230X-12-170

**Published:** 2012-11-29

**Authors:** Irbaz Bin Riaz, Haris Riaz, Talha Riaz, Sophia Rahman, Muhammad Amir, Maaz B Badshah, Abdul Nafey Kazi

**Affiliations:** 1 Department of Internal Medicine, University of Arizona, Tucson, AZ, USA; 2 Dow University of Health Sciences, Civil Hospital Karachi, Karachi, Pakistan; 3 New York Methodist Hospital, New York, NY, USA; 4 Albert Einstein College of Medicine, New York, NY, USA; 5 James J. Peters VA Medical Center/Mount Sinai School of Medicine, New York, NY, USA

## Abstract

**Background:**

Hepatocellular cancer is notorious for recurrence even after curative therapy. High recurrence determines the long term prognosis of the patients. Vitamin K2 has been tested in trials for its effect on prevention of recurrence and improving survival. The results are inconclusive from individual trials and in our knowledge no systematic review which entirely focuses on Vitamin K2 as a chemo preventive agent is available to date. This review is an attempt to pool all the existing trials together and update the existing knowledge on the topic.

**Methods:**

Medline, Embase and Cochrane Register of Controlled trials were searched for randomized controlled trials where vitamin K2 or its analogues, in any dosage were compared to placebo or No vitamin K2, for participants of any age or sex. Reference lists and abstracts of conference proceedings were searched by hand. Additional papers were identified by a manual search of the references from the key articles. Attempt was made to contact the authors of primary studies for missing data and with the experts in the field.

Trials were assessed for inclusion by two independent reviewers. Primary outcomes were recurrence rates and survival rates. There were no secondary outcomes. Data was synthesized using a random effects model and results presented as relative risk with 95% Confidence Intervals.

**Result:**

For recurrence of hepatocellular cancer after hepatic resection or local ablative therapy, compared with controls, participants receiving Vitamin K2, pooled relative risks for hepatocellular cancer were 0.60; 95% CI: 0.28–1.28, p = 0.64) at 1 yr 0.66; 95% CI: 0.47–0.91), p = 0.01) at 2 yr; 0.71; 95% CI: 0.58–0.85, p = 0.004) at 3 yr respectively. The results were combined using the random analysis model.

**Conclusion:**

Five RCTs evaluated the preventive efficacy of menatetrenone on HCC recurrence after hepatic resection or local ablative therapy. The meta-analysis of all five studies, failed to confirm significantly better tumor recurrence- free survival at 1 year. Improved tumor recurrence at 2nd and 3rd year may be just due to insufficient data. There was no beneficial effect on the overall survival. However, to confirm the beneficial effect or lack of it, large, higher quality randomized controlled trials are still required.

## Background

Hepatocellular carcinoma (HCC) is a significant global cause of morbidity and mortality. Overall, it is the third leading cause of the cancer death across the globe with numbers reaching 600,000 per year [1]. Long term prognosis remains poor even after the potentially curative treatments such as curative resection and radiofrequency ablation. Despite advancements in the curative treatments, the 5 year survival rate is only 50–70% [2]. Liver cirrhosis is considered as the most significant risk factor accounting for as much as 90% cases [3]. HCC is a rising global health problem, with incidence increasing in Asia, Europe and United States [2,4]. Although high recurrence of HCC is the major challenge but variable degree of benefit has been observed by use of chemotherapeutic agents such as interferon [5,6]. However, adverse effects such as bone marrow suppression after long term administration as well as the fact that beneficial effect is limited to virological responders limits its usefulness [7,8]. Currently, there is no single approved agent to prevent recurrence of HCC after the curative treatment.

HCC, just like other cancers require angiogenesis make it a target for anti angiogenic therapy [9]. This approach has proven successful at least in some animal models. The suppression of angiogenic pathways signaling pathways in murine models retards the HCC growth [10]. Drugs such as Sorafenib have not been able to establish themselves because of the costs and severe adverse effects profile [11,12]. Use of Vitamin K2 as an anti angiogenic factor is a potential therapy to prevent recurrence of HCC. Vitamin K2 is a fat soluble vitamin that is required as a co enzyme for the carboxylase involved in the posttranscriptional modification of clotting factors [13]. Abnormal uncarboxylated prothrombin (des-gamma-carboxy-prothrombin[DCP]) is found to be elevated in some patients of HCC with the aggressive phenotype. Vitamin K has been show to suppress plasma DCP concentrations. Vitamin K2 has been employed in several studies to delay and/or prevent the recurrence of HCC. It acts by suppressing the CyclinD1 expression through inhibition of NF Kappa B, thereby inhibiting the growth of HCC cells [14].

Several clinical trials have attempted to elucidate the role of Vitamin K2 in preventing the recurrence of HCC. The results are conflicting, some suggesting benefit while others failing to do so [1,3,15]. The individual trials are small and therefore underpowered. The aim of this systematic review is to identify the randomized clinical trials comparing vitamin K2 versus placebo (or no Vitamin K2) assessing the effect of Vitamin K2 on the recurrence of hepatocellular cancer once the curative treatment has been undertaken.

## Methods

### Study selection

**Figure 1:**
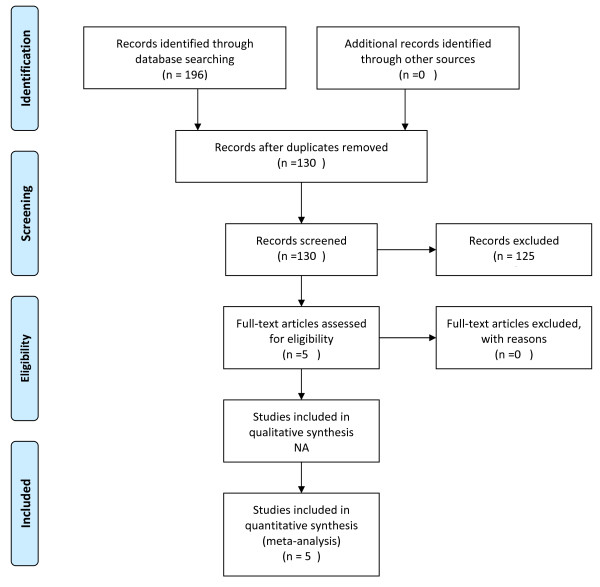
**Flow diagram highlighting the search strategy.** Selection process for studies included in meta analysis.

We searched PubMed (MEDLINE), EMBASE and Cochrane database of Controlled Trials from the 1990 to December 2011. Following terms as Medical Subject Headings and keywords were used; Vitamin K, Hepatocellular Cancer, Hepatectomy, Chemoprevention and Recurrence. Search Strategy is outlined as a flow sheet in Figure 1. Initially, No language restrictions were applied. Searches were limited to randomized clinical trials in human participants of any age or gender. A manual examination of references in selected articles was also performed. We also looked the websites pertaining to the clinical trials public registries (ClinicalTrials.gov and ClinicalStudyResults.org). The United State Food and drug Administration Website was searched for submissions pertaining to our concerned topic. We tried to contact the experts in the fieldbut the success was very limited. Eventually, articles in only English language were considered because of limited resources. The titles and abstracts of 5 potentially relevant references were identified through the literature search and reviewed independently by 2 investigators (R.I.B., R.H) to determine whether they met eligibility criteria for inclusion. Discrepancies regarding whether to include or exclude a study were resolved by discussion. Randomized clinical trials were included irrespective of publication status. Studies were eligible for inclusion if they were randomized controlled trials of Vitamin K2 treatment among persons who were considered cured after curative therapy. Studies were excluded if they were observational studies or recurrence events were not reported as an outcome at 12 months or if the participants were not disease free after the primary treatment of hepatocellular cancer. Search strategies for Medline and Cochrane database of controlled trials are attached as Appendix 1.

### Data abstraction

All data were independently abstracted by 2 investigators (R.I.B, R.H) using a standardized data collection form. Discrepancies were resolved through discussion among the investigators (R.I.B,R.H) and through reference to the original articles. We attempted to contact study authors for missing information when necessary, but the success was limited. Trial characteristics abstracted included design of the randomized controlled trial, type and number of control, number of treatment groups, description of treatment regimens (45 mg vs 90 mg), description of inclusion and exclusion criteria, and demographic characteristics of study populations at baseline. Some of the information is presented (Table 1). The outcomes recorded included the recurrence of hepatocellular cancer and the survival at 1,2 and 3 years post resection (Table 2). Data was either directly obtained from the trials or was calculated from the information available. Only Important information is presented in Table 2. Data was pooled for recurrence and survival at 1, 2 and 3 years by random effects Model.

**Table 1:** Baseline characteristics of study population

**Study**	**n**	**Age (Years)**	**Viral Status (C/B/C+B)**	**Alcohol Intake**^**@**^**(No/Yes)**	**Tumor Size (mm)**	**Tumor no**	**Child Pugh (A/B)**	**Treatment (Surgery/Local)**	**Follow up**
Mizuta et al.									
Treatment	32	63.3 ± 7.5	28/3/1	10/22	17.7 ± 5.1	1.5 ± 0.9	26/6	1/31	28.9 (± 8.3)
Control	29	64.5 ± 6.7	26/2/1	3/26	19.4 ± 6.9	1.5 ± 0.7	22/7	3/26	27.7 (± 8.6)
Kakizaki et al.									
Treatment	30	69.1 ± 5.9	30/0/0	6/24	20.4 ± 11.6	19/11	22/8	4/26/0	36
Control	30	69.0 ± 7	30/0/0	3/27	25.0 ± 9.4	22/8	22/8	7/23/0	36
Hotta et al.									
Treatment	21		14/6		18/3*	15/6	15/6	2/19	
Control	21		3/19		18/6*	12/12	12/12	2/22	
Yoshiji et al.									
Treatment	18*	62.8 ± 7.4	15/0	8/10	17.9 ± 9.2	1.6 ± 0.9	16/2	0/18	36
Control	25	60.5 ± 8.5	1/3	12/13	18.7 ± 9.5	1.6 ± 0.9	20/5	0/25	36
Yoshida et al.									
Treatment^#^	367	68.6 ±	305/38	140/227	19.9 ± 7.6	1.4 ± 0.7	323/44	14/353	37
Control	181	7.9	150/20	79/102	20.3 ± 7.6	1.4 ± 0.7	154/27	7/174	37

*The data in the treatment group of Yoshiji et al. is restricted to 18 patients to exclude 19 patients who received ACE-inhibitors and 25 patients who received a combination of ACE-Inhibitors and Vitamin K2.

@Mizuta et al. and Yoshiji et al. have defined heavy alcoholism by consumption greater than 40 grams/day whereas Kakizaki et al. has defined it as greater than 65 gm/dl. Yoshida et al. has defined at as consumption greater than thrice days per week regardless of the quantity.

#The treatment group in the study of Yoshida et al. compromised 367 patients, of whom 182 received drug at a dosage of 45 mg/day whereas the remaining 185 at 90 mg/day. The dosages in all the other studies is 45 mg/day.

**Table 2:** Summary of outcomes used as primary endpoint

**Study**	**Recurrence Rate %**			**Survival %**		
	**1 yr**	**2 yr**	**3 yr**	**1 yr**	**2 yr**	**3 yr**
Mizuta et al.						
Treatment	12.5	39	64.3	100	96.6	87
Control	55.2	83.2	91.6	96.4	80.9	64
Kakizaki et al.						
Treatment	7.7	51.4	61.2	100	95	77.5
Control	55.2	83.2	91.6	95.8	90.2	66.4
Hotta et al.						
Treatment	23.8	28.6	-	100	100	-
Control	33.3	46.5	73.3	87.5	81.7	81.7
Yoshiji et al.						
Treatment	22	44	61	100	94.4	88.9
Control	24	48	68	100	92	88
Yoshida et al.						
Treatment	28.2	-	-	97.2	-	-
Control	34.5	-	-	99.0	-	-

### Quality assessment

Two authors (R.I.B., R.H.) independently evaluated quality of each study using an established tool [16]. Nine domains were assessed: randomization, concealment of treatment allocation, similarity of groups at baseline, eligibility criteria, blinding of outcome assessor, patient and care provider, avoidance of co interventions and intention-to-treat analysis (Table 3). Disagreement was resolved through consensus and discussion. All studies reporting the recurrence as an outcome consistently were included in the final analysis.

**Table 3:** Summary of methodological assesment

	**Mizuta et al.**	**Yoshida et al.**	**Yojishi et al.**	**Kakizaki et al.**	**Hotta et al.**
Randomization*	No	Yes	Yes	No	Yes
Concealment to Treatment Allocation	No^@^	Yes	Yes	No^@^	Unclear
Avoidance of co-interventions	Yes	Yes^#^	Yes^%^	Yes	Yes
Similarity of groups at baseline	No^+^	Yes^&^	Yes	Yes	Yes
Eligibility Criteria	Yes	Yes	Yes	Yes	Yes
Blinding methods	No	Yes Invesitgator, study sponsor and patients	Yes	No	Unclear
Intention to Treat Analysis	Yes	Unclear	Unlcear	Unlcear	Unclear

*From Randomization, we mean whether the randomization methods have been described in methods as all these are RCT’s.

^@^We presume that the treatment allocation was not concealed as the control groups did not receive placebo.

#Use of glycirrhizic acid for the treatment of chronic hepatitis was not considered as an intervention.

&Only the difference in AST levels was statistically significant.

+The difference in the PIVKA 2 levels in baseline groups was statistically significant.

^%^We do not consider use of ACE-Inhibitors as a co-intervention as we have excluded those patients from the analysis.

### Statistical analysis

Results are reported as Relative Risks with 95% confidence intervals calculated. Heterogeneity is assessed by the inspection of the forest plot, and using *X*^2^ tests with n-1 degree of freedom where n=number of studies involved. A P value of 0.10 is defined as significant. The I^2^ statistic is also inspected with values greater than 50% consistent with significant heterogeneity.

It was expected a priori difference in outcome may be contributed by many factors including the cause of hepatocellular cancer, dosage of Vitamin K2 used, method used for the curative resection and age and sex of the patient. However no subgroup analysis was performed because of the small size and number of studies. Moreover, no formal assessment of publication bias by funnel plot was done because of small number of included studies. However we conducted sensitivity analysis for recurrence at 1 year by excluding the study by Yojishi and colleagues as it tested the effect of combination therapy and also by excluding the study by Yoshida and colleagues which is by far the largest trial.

## Results

Of the 5 potentially relevant studies identified in the initial literature search, 4 were included in the meta-analysis. Another study was identified by the manual search of the references of the key articles,making it a total of 5 studies (Figure 1). The baseline characteristics of participants such as the frequency of viral hepatitis B and C, alcohol intake, tumor size, number of tumors, child pugh classification differed among the participants of the studies. Table 1 describes the baseline characteristics of participants included in the study. Table 2 summaries the results for the both the primary outcomes of the study. Table 3 describes the quality assessment characteristics of trials included in the meta-analysis.

The dose of Vitamin K2 administered varied between studies, either 45 mg/d or 90 mg/d. Follow up in all studies included was done at 12 ,24 and 36 months except the study done by Yoshida and colleagues [1]. Entry criteria also varied between studies; however, all studies required the patients to be cured after the primary treatment of hepatocellular cancer.

**Figure 2:**
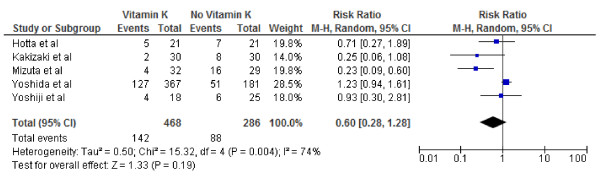
**Effect of Vitamin K**_**2**_**on 1-year tumor recurrence after curative therapy for hepatocellular carcinoma.**

**Figure 3:**
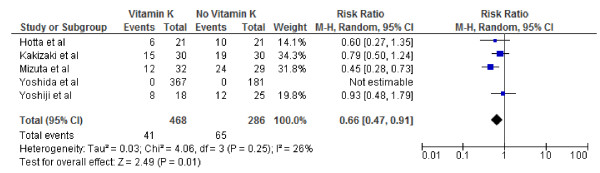
**Effect of Vitamin K**_**2**_**on 2-year tumor recurrence after curative therapy for hepatocellular carcinoma.**

**Figure 4:**
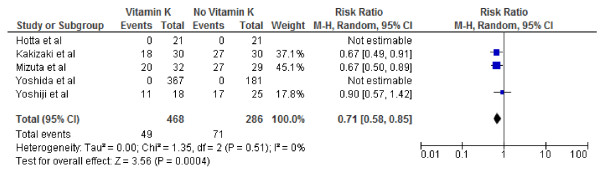
**Effect of Vitamin K**_**2**_**on 3-year tumor recurrence after curative therapy for hepatocellular carcinoma.**

**Figure 5:**
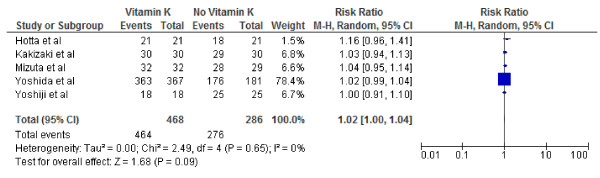
**Effect of Vitamin K**_**2**_**on 1-year survival after curative therapy for hepatocellular carcinoma.**

**Figure 6:**
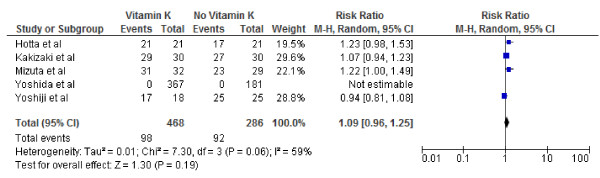
**Effect of Vitamin K**_**2**_**on 2-year survival after curative therapy for hepatocellular carcinoma.**

**Figure 7:**
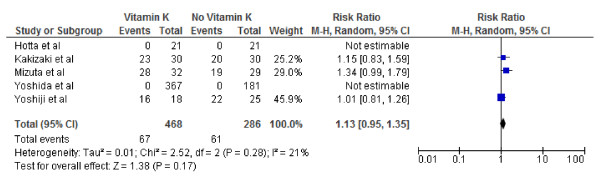
**Effect of Vitamin K**_**2**_**on 3-year survival after curative therapy for hepatocellular carcinoma.**

The 5 studies included in the meta-analysis incorporated data from 754 participants who were free of hepatocelluar cancer after the primary treatment. The cause of hepatocelluar cancer varied between studies (Table 1). Pooled overall RRs are presented for recurrence and survival of hepatocellular cancer Figures 2, 3, 4, 5, 6, and 7.

Three earlier studies showed significantly better tumor recurrence-free survival, while all the studies failed to show any significant effect on overall survival. However, the largest and most recent trial by Yoshida and colleagues enrolling 548 patients (or 72.7% patients of this meta-analysis) failed to confirm the beneficial effect on tumour recurrence-free survival.

One of the first randomized controlled trials on the topic was a pilot study done by Mizuta and colleagues [15]. Study included 61 patients and suggested the beneficial effect of Vitamin K2 both on the recurrence as well as survival. The cumulative recurrence rates in the treatment group were 12.5% at 1 year, 39.0% at 2 years, and 64.3% at 3 years; the corresponding recurrence rates in the control group were 55.2%, 83.2%, and 91.6%, respectively. The results were statistically significant. The 1-year, 2-year and 3-year cumulative overall survival rates for the treatment group were 100%, 96.6% and 87%, respectively, while the 1-year, 2-year, and 3-year cumulative survival rates for the control group were 96.4%, 80.9% and 64%, respectively. These results were not statistically significant. In another study conducted by Hotta and colleagues [17] the cumulative incidence of HCC recurrence did not differ between the two groups, 33% and 50% in the treatment and control respectively, and the cumulative overall survival rate tended to be higher in the treatment group. However, this result was not statistically significant. Kakizaki and colleagues in a study showed that vitamin K2 had a suppressive effect on the recurrence of HCC, but there was no significant difference in the overall survival rates [3]. The cumulative recurrence-free rates in the vitamin K2 group were 92.3% at 1 year, 48.6% at 2 years and 38.8% at 3 years; those in the control group were 71.7%, 35.9% and 9.9%, respectively. The difference was significant. The cumulative overall survival rates in the vitamin K2 group were 100% at 1 year, 95.0% at 2 years and 77.5% at 3 years, and the corresponding rates in the control group were 95.8%, 90.2% and 66.4%, respectively. There was no significant difference between the two groups. In the trial conducted by Yojishi and colleagues, a combination treatment with a vitamin K2 analogue (menatetrenone, 45 mg/day) and an angiotensin converting enzyme inhibitor (ACE-I) (perindopril, 4 mg/day) for 36–48 months after curative therapy for HCC markedly inhibited the cumulative recurrence of HCC, as well as suppressed of the serum levels of the vascular endothelial growth factor [4]. The serum levels of lectin-reactive alpha-fetoprotein were also suppressed in addition and almost in parallel with vascular endothelial growth factor.

In a Recent Trial conducted by Yoshida and colleagues [1] a total of 548 patients were enrolled at 31 study sites in Japan and randomly assigned between March 2004 and September 2005. HCC recurrence (i.e., intrahepatic lesions adjacent to or distant from previously treated nodules, and extrahepatic metastasis), cancer other than HCC, or death from any cause were detected in 58, 52, and 76 patients in the placebo,45-mg/day, and 90-mg/day groups respectively. This trial also assessed the safety of Vitamin K2 administration as well as the dose response relationship. While conducting the Meta analysis both 45 mg/day as well as 90 mg day arm were combined into a single treatment group.

We conducted both fixed and random effect meta-analysis on these five studies to assess the effects of vitamin K2 on tumour recurrence rates and patient survival. Figures 2, 3 and 4 show the pooled RR of 1-year, 2-year and 3-year tumour recurrence after curative therapy in the vitamin K2 treatment patients versus no treatment patients.

Only results from random effects Model are reported because of significant heterogeneity.

Because of the lack of data on 3-year recurrence in the study by Hotta and colleagues and lack of data on 2nd and 3rd year recurrence in the study by Yoshida and colleagues the meta analysis for 2 and 3 year recurrence included 4 and 3 studies respectively.

Our meta-analysis by random effects model effect model showed that vitamin K2 did not significantly decreased HCC recurrence rates at 1 year, however it did appear to significantly decreased HCC recurrence rates 2 and 3 years after potentially curative therapy: 1-year tumor recurrence (RR: 0.60; 95% CI: 0.28–1.28, p = 0.64); 2-year tumor recurrence (RR: 0.66; 95% CI: 0.47–0.91), p = 0.01); 3- year tumor recurrence (RR: 0.71; 95% CI: 0.58–0.85, p =0.004). I^2^ values were calculated to quantify heterogeneity between studies The ^I^ values were 74% (P = .004) and 26% (P = .25)and 0% P = .51) for recurrence at 1,2and 3 years respectively, indicating significant heterogeneity between studies Included for analysis at 1 year and no heterogeneity between studies used for recurrence at 3 years. Vitamin K2 did significantly decreased HCC recurrence rates at 1 year if study by Yoshida and colleagues was excluded (RR: 0.46; 95% CI: 0.22–0.94), p = 0.03). The ^I^ values also came down form were 74% (P = .004) to 41% (P = .016) hence the exclusion explaining some heterogeneity. However, the effect of Vitamin K2 failed to become significant 1 year if the study by Yojishi and colleagues were excluded ruling out any possible influence of combination therapy (Vitamin K2 and ACE inhibitor) on the overall result.

Figures 5, 6 and 7 show Forest plots of vitamin K2 on the 1, 2- and 3-year survivals following curative therapy using the random effect model. As indicated, vitamin K2 did not significantly increase overall survival at either 1 (RR: 1.02; 95% CI: 1.00–1.04, p = 0.09), 2-year(RR: 1.09; 95% CI: 0.96–1.25, p = 0.19) or 3-year(RR: 1.13; 95% CI: 0.95–1.35, p = 0.17) survival. I^2^ were calculated as 0% (P = .65), 59% (P = .06), 21% (P = .28) indication significant, no and mild heterogeneity respectively.

## Discussion

This meta-analysis is unique in that, to our knowledge, it is the first to focus entirely on the role of vitamin K2 in the prevention of hepatocellular carcinoma after the curative therapy for hepatocellular carcinoma. Previously Chu and colleagues investigated the role of Vitamin analogues in chemoprevention of hepatocellular carcinoma in a meta-analysis [18]. The study suggested that there is evidence to suggest that chemo-preventive therapy after partial hepatectomy and local ablative therapy in the form of vitamin analogues is beneficial in prolonging the disease free survival, but evidence is less for an effect of overall survival. The meta analysis was based on a 4 studies with a very small number of patients. Our meta analysis includes a recently published RCT by Yoshida and Colleagues [1] which is a relatively large trial including 548 patients (or 72.7% patients of this meta-analysis). It has been suggested in the past that Vitamin K2 may be useful in preventing both the early and late type of recurrences [15] possibly by inhibiting the proliferation of HCC cells through cell cycle arrest at the G1 phase or via suppression of cyclin D1 expression through the IKK/IkappaB/nuclear factor-kappaB pathway [19,20]. However, the present meta analysis fails to confirm the benefit of Vitamin K2 in reducing the recurrence and increasing the disease free survival after the curative treatment of HCC. All trials were conducted in japan and only one trial was multi -centered. The quality of trials was variable (Table 3). It was difficult to determine precisely the quality of included trials as many omitted important methodological details.

There are several limitations inour study. It is meta analysis of a small number of trials. The recurrence and survival data in the largest trial by Yoshida et al. [1] was available for only 1 year and not 2 and 3 year time. Hence the results are limited for the recurrence and survival at 2nd and 3^rd^ years. Moreover in the original trial the treatment groups were divided into two groups 45 mg/day and 90 mg/day but for the purpose of our analysis we considered both groups as a single treatment group. However due to s small sample size we could not separately assess the effect of different doses of Vitamin K2. Moreover, for several outcomes the heterogeneity was significant e.g. for recurrence at 1 year I2 = 74 indicating significant heterogeneity making the results less reliable. One of the included studies done by Yoshiji and colleagues tested a combination of Vitamin K2 and ACE inhibitor. We also pooled the results without the former but the still the results for recurrence at I year were unchanged.

In future there is a necessity to conduct larger trials to confirm or decline the benefits of Vitamin K2 agonists in reference to hepatocellular carcinoma. With larger patient numbers it may be possible to perform the subgroup analysis to evaluate for heterogeneity of of studies. We also suggest the authors of future trials to follow consort statement for better methodological assessment of trials. It may be beneficial if the trials are also conducted in other parts of the world as so far all the existing knowledge on the topic comes from Japan.

## Conclusions

This meta-analysis of five randomized controlled trials, failed to confirm significantly better tumor recurrence- free survival at 1 year. Improved tumor recurrence at 2nd and 3rd year may be just due to insufficient data in terms of small sample sizes of the included articles. There was no beneficial effect on the overall survival. We therefore conclude that there is insufficient evidence at present to recommend administration of vitamin K2 for preventing the recurrence of hepatocellular carcinoma after curative resection. However, to confirm the beneficial effect or lack of it, large, higher quality randomized controlled trials are still required.

## Appendix 1

Database: Ovid MEDLINE(R) <1948 to November Week 3 2011>

Search Strategy:

1 exp Carcinoma, Hepatocellular/ (51694)

2 Post resection.mp. orexp Neoplasm Recurrence, Local/ (73315)

3 expHepatectomy/ (19379)

4 Vitamin K.mp. (13605)

5 exp Chemoprevention/ (10636)

6 exp Recurrence/ or exp Neoplasm Recurrence, Local/ or recurrence.mp. (302425)

7 randomized controlled trial.pt. (322599)

8 controlled clinical trial.pt. (84057)

9 randomized.ab. (227373)

10 placebo.ab. (130354)

11 clinical trials as topic/ (159645)

12 randomly.ab. (163568)

13 trial.ti. (97823)

14 7 or 8 or 9 or 10 or 11 or 12 or 13 (749301)

15 1 or 2 or 3 (138152)

16 4 or 5 (24233)

17 6 and 14 and 15 and 16 (21)

18 from 17 keep 1,3,7-9 (5)

Database: EBM Reviews - Cochrane Central Register of Controlled Trials <4th Quarter 2011>

Search Strategy:

1 exp Carcinoma, Hepatocellular/ (648)

2 Post resection.mp. (17)

3 expHepatectomy/ (316)

4 1 or 2 or 3 (882)

5 Vitamin K.mp. (492)

6 exp Chemoprevention/ (937)

7 exp Recurrence/ or exp Neoplasm Recurrence, Local/ (12338)

8 5 or 6 (1429)

9 4 and 7 and 8 (4)

## Competing interests

The authors declare that they have no competing interests.

## Authors’ contributions

IBR and HR conceived the study and did the literature search and quality assessment. IBR performed the statistical analysis. All the authors were involved in manuscript writing and preparation of the manuscript. All authors have read and approved the final manuscript.

## Pre-publication history

The pre-publication history for this paper can be accessed here:

http://www.biomedcentral.com/1471-230X/12/170/prepub
